# Assessment of salivary miRNA, clinical, and demographic characterization in colorectal cancer diagnosis

**DOI:** 10.1016/j.tranon.2024.101880

**Published:** 2024-01-22

**Authors:** Maryam Koopaie, Soheila Manifar, Mona Mohammad Talebi, Sajad Kolahdooz, Amirnader Emami Razavi, Mansour Davoudi, Sara Pourshahidi

**Affiliations:** a Department of Oral Medicine, School of Dentistry, Tehran University of Medical Sciences, Tehran, Iran; b Department of Oral Medicine, Imam Khomeini Hospital, Tehran University of Medical Sciences, Tehran, Iran; c Universal Scientific Education and Research Network (USERN), Tehran, Iran; d Iran National Tumor Bank, Cancer Research Center, Cancer Institute of Iran, Tehran University of Medical Sciences, Tehran, Iran; e Department of Computer Science and Engineering and IT, School of Electrical and Computer Engineering, Shiraz University, Shiraz, Iran; f Department of Oral and Maxillofacial Medicine, School of Dentistry, Tehran University of Medical Sciences

**Keywords:** Colorectal cancer, microRNA, Saliva, Clinical characterizations, Machine learning

## Abstract

**Aim:**

Colorectal cancer (CRC), as the third most frequent malignancy in the world, is the fourth major cause of cancer-related mortality. Its early detection contributes significantly to a reduction in mortality. The objective of this case-control research was to analyze the salivary expression of microRNA-29a (miR-29a) and microRNA-92a (miR-92a), and also to consider demographic, clinical, and nutritional habits for differentiation between CRC patients and healthy controls, especially in the early stages.

**Method:**

A standard checklist was used to obtain the demographic information, clinical features, and dietary habits of the case and control groups. Samplings of whole unstimulated saliva samples were obtained from 33 healthy persons and 42 CRC patients. Through real-time PCR, statistical analyses, and machine learning analyses, miR-29a and miR-92a salivary expression levels were evaluated.

**Results:**

The mean salivary expression of miR-92a and miR-29a in CRC patients was significantly higher than in healthy controls (*p* < 0.001). The area under the receiver operating characteristic curve for miR-92a and miR-29a salivary biomarkers was 0.947 and 0.978, respectively. The sensitivity and specificity values for miR-92a were 95.24 % and 84.85 %, respectively, whereas sensitivity and specificity for miR-29a were equal to 95.20 % and 87.88 %, respectively. Multiple logistic regressions considering demographics, clinical features, and nutritional habits led to values of 95.35 % and 96.88 % as sensitivity and specificity, respectively, and machine learning analysis led to values of 88.89 % and 86.67 % as sensitivity and specificity, respectively.

**Conclusion:**

CRC could be accurately diagnosed based on miR-92a and miR-29a levels in saliva. Statistical analysis and machine learning might develop cost-effective models for the distinction of CRC using a noninvasive technique.

## Highlights

• Saliva-based examination of miR-92a and miR-29a levels can be used for the early diagnosis of colorectal cancer.
• Diagnostic accuracy could be enhanced by simultaneously including demographic and clinical characteristics.
• Statistical analysis and machine learning might develop cost-effective models for distinguishing CRC using a noninvasive technique.

## Graphical abstract

**Figure:**
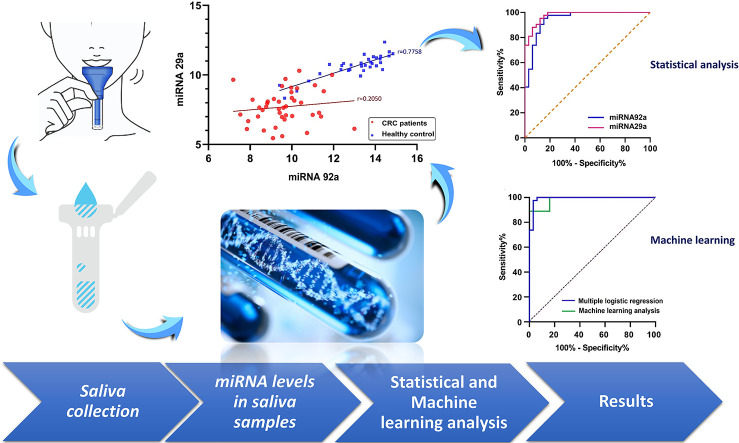

## Introduction

As the third most prevalent malignancy worldwide, colorectal cancer (CRC) is the fourth leading cause of cancer-related mortality [1]. Geographic location and regional culture influence the incidence of CRC [2], and environmental and genetic factors contribute to CRC development. Diet (especially intake of animal fat and nitrites, and inadequate intake of fruits, fiber, and vegetables), obesity, physical inactivity, and cigarette and alcohol use are modifiable lifestyle risk factors for CRC [3,4].

Some nonmodifiable risk factors include age older than 50 years, history of malignancy, and genetic predisposition [5]. Screening for and diagnosis of CRC in the initial stages are critical for reducing mortality rates [6]. Colonoscopy is the most common method for CRC screening and has the highest sensitivity and specificity of all CRC screening tools. Important drawbacks of colonoscopy include operator dependence, as seen by the inverse correlation between adenoma detection rate and post-colonoscopy CRC risk; considerable patient burden; and high expense [5,7].

The fecal occult blood test (FOBT) is recommended as a population-based screening tool for CRC; however, this test is not conducted to identify CRC precursor lesions but would be continued at timed periods to enhance sensitivity [8]. The other screening tool for CRC is flexible sigmoidoscopy. However, some complications, such as sigmoid perforation and bleeding, can occur [9]. These methods not only are invasive but also lack the sensitivity and specificity necessary for accurate diagnosis. Therefore, some noninvasive methods, such as examination of the expression of CRC biomarkers, require further study [10,11].

MicroRNAs (miRNAs) are noncoding RNAs that have essential post-transcriptional regulatory functions [12]. miRNAs have a significant role in carcinogenesis through oncogenic or tumor-suppressive activity [13]. Circulating miRNAs can be detected in plasma, serum, saliva, urine, and other body fluids. Collecting saliva instead of a blood sample has considerable advantages because of its noninvasive procedure, ease of storage, and cost-effectiveness [14]. Recent studies suggest a correlation between salivary miRNAs and malignancies outside of the oral cavity [15], [16], [17]. miR-92a and miR-29a are highly up-regulated in the plasma of patients with CRC, according to previous research, and can possibly be used as noninvasive molecular markers for CRC detection [18], [19], [20].

Studies have reported that miR-92a plays a positive role in colorectal carcinogenesis by promoting the proliferation and migration of CRC cells by targeting several well-studied genes, including KLF4, PTEN, and DKK3 [21,22]. MiR-92a is up-regulated in tumor tissues of CRC patients compared with adjacent normal tissues [21]. Yang et al. suggested that miR-92a was significantly differentially expressed between CRC patients and healthy controls [19]. Overall, miR-92a has been shown to promote the growth and migration of CRC cells and may serve as a potential biomarker for diagnosing CRC. In addition, the study found that plasma miR-29a has strong potential as a novel noninvasive biomarker for the early detection of CRC [23]. miR-29a has been reported to promote CRC cell invasion by regulating the expression of matrix metalloproteinase 2 (MMP2) and E-cadherin by targeting KLF4 [24]. KLF4 was identified as a direct target gene of miR-29a, and miR-29a promoted CRC cell invasion, which was blocked by the re-expression of KLF4.

We investigated the potential for using saliva miRNAs for the early diagnosis of CRC as a noninvasive diagnostic tool. The purpose of this research is to examine the use of salivary levels of miRNAs in CRC diagnosis, taking into account the relevance of early detection of CRC by noninvasive approaches. Furthermore, based on salivary miRNA levels, demographic data, clinical features, and food consumption patterns, a CRC detection algorithm was built using statistical and machine learning methodologies.

## Methods

### Ethical statement

This study was authorized by the Tehran University of Medical Sciences’ Ethical Committee (ethical code: IR.TUMS.DENTISTRY.REC.1399.084). Before participating in this study, all participants completed an informed consent form that detailed the study's aims. All procedures were carried out under the relevant guidelines and manufacturer's instructions for each instrument.

### Samples

The case-control research included 34 healthy people and 42 CRC patients at various stages (Cancer Institute of Imam Khomeini Hospital, Tehran, Iran). Gastroenterologists diagnosed them with CRC based on histopathological and colonoscopy examinations. Subjects were excluded based on the following criteria: (1) people suffering from active dental and periodontal diseases; (2) people with a history of previous malignancies, as well as any obvious inflammatory conditions or metabolic abnormalities such as (3) people with a history of surgery and cancer treatments; (4) individuals who had received blood during the past 3 years; and (5) women who were pregnant.

**Fig. 1:**
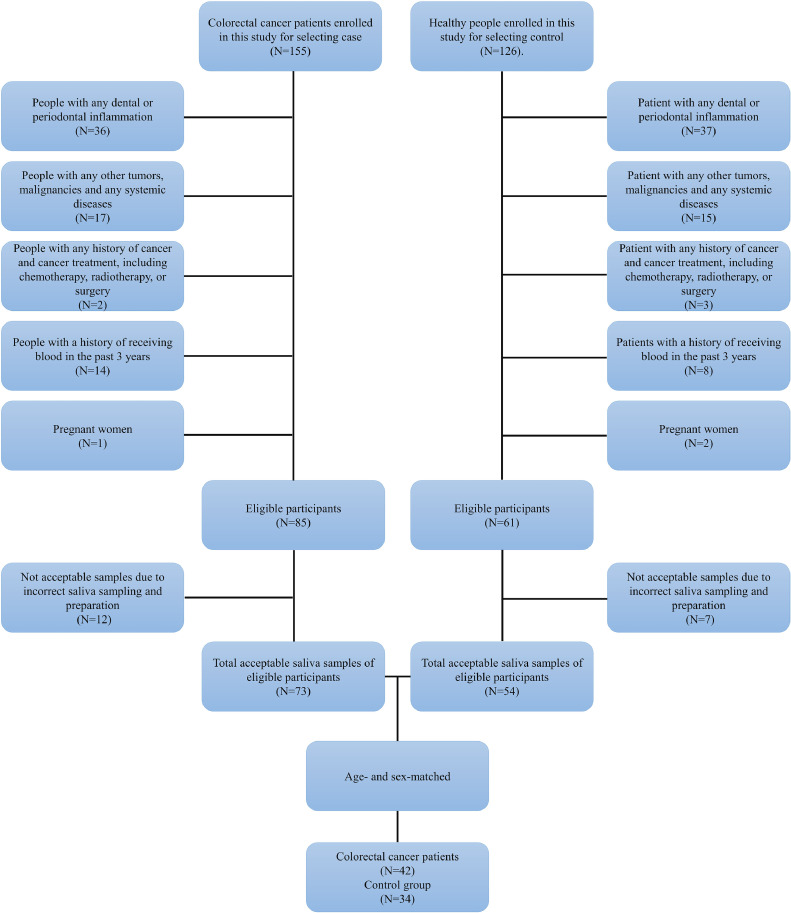
The STARD diagram (CRC patients and controls).

The control group consisted of healthy patients referred to Imam Khomeini Hospital in Tehran for regular medical examinations and dental checkups. Likewise, the excluded criteria for the case group was used for control groups. CRC patients and controls were age- and gender-matched (Fig. 1). To define their demographic data, all participants were asked to fill out a standardized checklist to gather information with regard to gender, age, body mass index (BMI), history of malignancy, education level, physical activity, food intake habits, alcohol consumption, and smoking, as well as behavior that may affect CRC progression. In addition, all individuals were asked whether they had a history of adenomatous polyposis or Crohn's disease, as well as a personal or family history of malignancy. Smoking status referred to current smokers who smoked at least one cigarette per day [25].

### Saliva collection

Saliva samples were taken between 8:00 and 10:00 am to avoid the probable impact of circadian rhythm. To prevent salivary irritation, individuals were requested to refrain from eating and smoking 1 h prior to saliva collection. Following dental and periodontal examinations, the spitting technique was used to collect whole unstimulated saliva. Saliva was collected for 5 to 10 min at 1-minute increments and poured into 10-ml sterilized Falcon tubes.

### MiR-29a and miR-92a levels in saliva samples



### Isolation of RNA

Saliva samples were used to extract the total RNA using Trizol reagent according to the manufacturer's instructions (RiboEx Kit; GeneAll, Korea). The purity of extracted RNAs was determined using a NanoDrop spectrophotometer and gel electrophoresis (Thermo Scientific, USA), and samples after elution were kept at –80 °C. Using ExoQuick precipitation techniques, exosomes were extracted from plasma samples. RNA was isolated from exosomes and plasma samples using a Qiagen miRNeasy Mini Kit.

### Synthesis of CDNA and RT-qPCR

The primers of miR-29a, miR-92a, and housekeeping genes (U6) was designed and synthesized by Pishgam Company (Iran). Table 1 contains a listing of designed and used primer sequences.

**Table 1:** RT-qPCR primer sequences of miR-29a and miR-92a.

**miRNA name**	**Primer name**	**Primer sequence (5′ to 3′)**
miR-29a	RT- primer	GTCGTATCCAGTGCAGGGTCCGAGGTATTCGCACTGGATACGATAACCG
	Forward	CGTAGCACCATCTGAAATCG
miR-92a	RT- primer	CTCAACTGGTGTCGTGGAGTCGGCAATTCAGTTGAGTCAGG CCG
	Forward	GCTGAGTATTGCACTTGTCCCG
U6	RT- primer	GTCGTATCCAGTGCAGGGTCCGAGGTATTCGCACTGGATACGACAAAATG
	Forward	GCGCGTCGTGAAGCGTTC
Primer	Universal Reverse	GTGCAGGGTCCGAGGT

Briefly, extracted total RNA was obtained using riboEX reagent (GeneAll Biotechnology, Korea). TaKaRa's RNA PCR Kit (TaKaRa, Bio, Shiga, Japan) was applied to synthesize cDNA by Parsgengan. Nano-Drop equipment (ND-2000, Thermo Scientific, USA) was used to assess the amount and purity of extracted RNA. CDNA was synthesized with a Universal cDNA Synthesis Kit (Parsgengan, Iran).

### Real-time quantitative reverse-transcription PCR

Using SYBR green/ROX (BioFACT, Daejeon, South Korea), the product was utilized for quantitative real-time PCR. Real-Time PCR Master Mix (A323402, Ampliqon, Stenhuggervej, Denmark) was utilized in conjunction with the Exicycler 96 Real-Time Quantitative LightCycler 480 System Instrument II protocol (Roche, USA). The amplification technique included one cycle at 95 °C for 1 min, followed by 40 cycles at 95 °C for 10 min and 60 °C for 40 s. Roche software (Roche Group, Basel, Switzerland) was used to calculate the cycle of threshold (CT). The delta threshold cycle value (ΔCT) for each sample was used to compute the expression of the investigated genes relative to the housekeeping gene; each experiment was conducted three times, and the mean value was recorded.

Ampliqon SYBR Green Master Mix (A323402, Ampliqon, Denmark) and miR-29a-specific primers (5′GTGCAGGGTCCGAGGT-3′) and housekeeping genes (U6) (5′GCGCGTCGTGAAGCGTTC-3′) were used in reverse transcription–quantitative real-time polymerase chain reaction (RT-qPCR). The mean value of reactions was reported after three replications. The RT-qPCR experiment was carried out as follows utilizing a Thermal Cycler (Qiagen, Rotor-Gene Q): one cycle of 15 min at 95 °C, followed by 40 cycles of 15 s at 95 °C, 30 s at 60 °C, and 20 s at 72 °C. The 2^–ΔΔCt^ technique was used to determine the relative expression levels of miRNAs in comparison with the levels of the U6 snRNA.

### Statistical methods

The RT-qPCR findings were analyzed using SPSS software v.25 (SPSS Inc., USA).miR-29a and miR-92a expression levels were compared between the case and control groups using the *t*-test. For examination of the receiver operating characteristic (ROC) curve to identify the optimal cutoff value, the Youden index was used, which is calculated as follows: *J* = sensitivity + specificity. The optimal cut-off point on the ROC curve maximizes the Youden index, corresponding to the point with the highest sum of sensitivity and specificity [26,27].

All findings are reported as mean ± SD (standard deviation), and a p-value less than 0.05 is deemed statistically significant. Using multiple logistic regression, the coefficients for predicting CRC were determined. To choose coefficients for the model, a stepwise forward regression approach was used.

### Machine learning method

To evaluate the effectiveness of miR-29a and miR-92a, besides other extracted features, in diagnosing CRC, we used logistic regression and multilayer feed-forward neural networks as two supervised machine learning methods, utilizing Python Software 3.7, the Keras library [28], and the Scikit-learn library. During the training phase, the extracted features were normalized as 0 and 1. The normalized values were entered into the logistic regression and neural network models. The output value represents the data sample's label. A total of 80 % of data samples were utilized for training and validation, whereas 20 % remained for the test phase. A fivefold cross-validation approach and Adam as the optimization algorithm were used. Samples of data were categorized as follows. Patients with CRC were classified as 1, whereas controls were labeled as 0. Each of the two hidden layers of the created model had 16 neurons. For the hidden layers, the ReLU activation function was used, whereas the sigmoid function was utilized for the output layer. As the loss function of the neural network, we also used binary cross-entropy.

## Results

### Characteristics of patients

In the CRC group, 19 patients had a positive family history of cancer. Table 2 provides a summary of the clinical and demographic data of CRC patients and controls. Cases and controls differed significantly based on BMI (*p* = 0.029), history of malignancy (*p* < 0.001), fast food consumption (*p* = 0.043), physical activity (*p* = 0.043), and consumption of sweet drinks (*p* = 0.014). However, there was no statistically significant difference in gender, age, current smoking status, alcohol consumption, meat consumption, or fruit and vegetable consumption between CRC patients and healthy controls (Table 2).

**Table 2:** Demographic and clinical data of case and control groups.

Characteristics	CRC patients (*n* = 42)	Healthy controls (*n* = 33)	p*
*Gender*			0.971
Female	19 (45.23 %)	18 (54.54 %)	
Male	23 (54.76 %)	15 (45.45 %)	
*Age, years*	54.12±13.98	42.33±12.50	0.392
*BMI*	26.37±3.22	25.64±4.45	0.029
*History of malignancy*	18 (42.86 %)	2 (6.06 %)	<0.001
*Physical Activity*			0.043
Low	4 (9.52 %)	17 (51.51 %)	
Intermediate	16 (38.09 %)	8 (24.24 %)	
High	22 (52.38 %)	8 (24.24 %)	
*Current smoking status*	10 (23.81 %)	5 (15.15 %)	0.061
*Alcohol consumption*	1 (2.38 %)	1 (3.03 %)	0.733
*Fast food consumption*			0.043
Don't use or less than one time per week	16 (38.09 %)	4 (12.12 %)	
One time per week	25 (59.52 %)	16 (48.48 %)	
More than one time per week	1 (2.38 %)	13 (39.39 %)	
*Meat*			0.083
Don't use or less than one time per week	8 (19.05 %)	4 (9.52 %)	
One time per week	25 (59.52 %)	15 (35.71 %)	
More than one time per week	9 (21.43 %)	14 (42.42 %)	
*Sweet drinks*			0.014
Don't use or less than one time per week	17 (40.47 %)	6 (18.18 %)	
One time per week	25 (59.52 %)	7 (21.21 %)	
More than one time per week	0 (0.0 %)	20 (60.60 %)	
*Fruits and vegetables*			0.257
Don't use or less than one time per week	19 (45.24 %)	6 (18.18 %)	
One time per week	16 (38.09 %)	12 (21.21 %)	
More than one time per week	7 (16.67 %)	18 (60.60 %)	
*Expression of miR-29a*	7.70±1.22	10.65±0.81	*p* < 0.001
*Expression of miR-92a*	9.64±1.21	13.09±1.43	*p* < 0.001
*TNM stage*			
I	5 (11.9 %)		
II	8 (19.05 %)		
III	14 (33.33 %)		
IV	15 (35.71 %)		
*Nodal status*			
Positive	29 (69.05 %)		
Negative	13 (30.95 %)		
*Tumor location*			
Rectum	22 (52.38 %)		
Distal colon	11 (26.19 %)		
Proximal colon	9 (21.43 %)		

Values are expressed as mean ± SD or No. (%).

⁎ *P*-value (p) for the comparison between CRC cases and controls.

### MiR-29a and miR-92a salivary expression

**Fig. 2:**
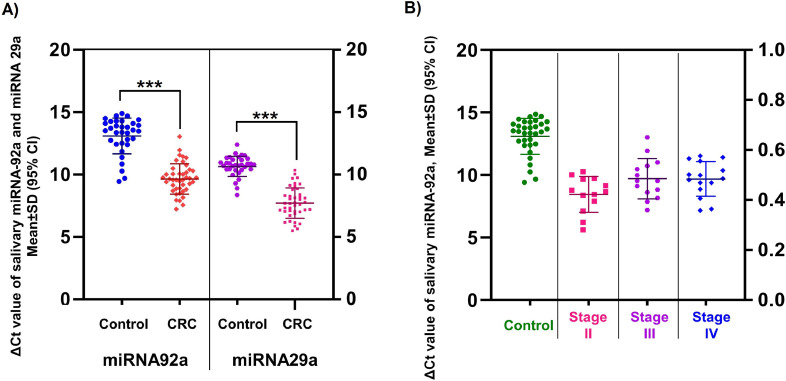
(A) Mean value of ∆Ct ± SD miR-92a and miR-29a in the saliva of CRC and controls, (B) Comparison of mean value of ∆Ct ± SD miR-92a in the saliva of CRC patients at various stages of CRC.*** *p* < 0.001.

The Ct-value of salivary miR-92a in CRC patients was 9.87 ± 1.91. In the healthy control group, this value was 13.14 ± 1.44. The Ct-values of salivary miR-29a were 7.98 ± 2.19 and 10.64 ± 0.79 for CRC patients and healthy controls, respectively. A *t*-test analysis showed that miR-29a and miR-92a expression were up-regulated significantly (decreasing ∆Ct and consequently increasing 2^–∆∆Ct^ and up-regulation in CRC patients) in saliva of CRC patients versus controls (*p* < 0.001; Fig. 2A).

Expression levels of miR-92a in various stages of CRC showed no significant difference in salivary samples; however, the differences between cases and controls were higher in early stages. This could provide hope for CRC diagnosis in the early stages (Fig. 2B).

**Fig. 3:**
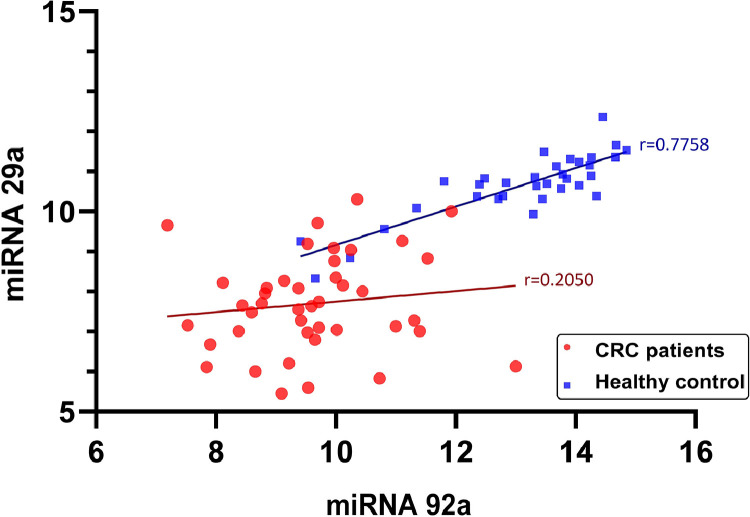
The correlations between miR-29a and miR-92a. *r* = 0.2050, *p* = 0.1927 for CRC patients and *r* = 0.7758, *p* < 0.0001 for healthy controls.

Pearson analysis revealed that there was a significant correlation between miR-92a and miR-29a salivary levels (*p* < 0.001, *r* = 0.819; Fig. 3).

**Fig. 4:**
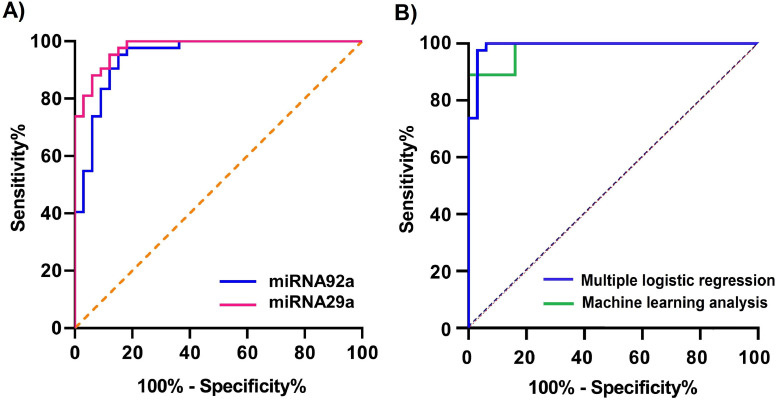
(A) ROC curve of salivary miR-92a and miR-29a in differentiation of CRC from healthy controls; (B) ROC curve for multiple logistic regression and machine learning analysis for discriminating CRC from healthy controls.

When 11.67 is considered as a cut-off value of ∆Ct, based on the Youden index method, the sensitivity and specificity of miR-92a salivary levels were 95.24 % and 84.85 %, respectively, and the sensitivity and specificity of miR-29a salivary levels in CRC diagnosis was 95.20 % and 87.88 %, respectively (cut-off value of ∆Ct = 9.820). The area under the curve (AUC) was 0.947 for miR-92a and 0.978 for miR-29a in CRC diagnosis compared with controls (Fig. 4A).

The association between miR-29a and miR-92a with all the clinical and demographic data of the case and control groups is summarized in Table 3. There is significant association of age, history of malignancy, and consumption of fast food, meat, and fruits and vegetables with both miR-92a and miR-29a, based on Spearman correlation analysis.

**Table 3:** Association between salivary levels of miR-92a and miR-29a with clinical and demographic data.

	**MiR-92a**		**MiR-29a**	
	**Spearman *r* (95 % CI)**	***p* (two-tailed)**	**Spearman *r* (95 % CI)**	***p* (two-tailed)**
**Age**	−0.368 (−0.554, −0.1473)	0.0012	−0.410 (−0.587, −0.195)	0.0003
**Gender**	−0.0942 (−0.321, 0.142)	0.4213	−0.029 (−0.261, 0.206)	0.8053
**BMI**	−0.0740 (−0.302, 0.162)	0.5278	−0.176 (−0.393, 0.059)	0.1303
**History of malignancy**	−0.386 (−0.569, −0.168)	0.0006	−0.386 (−0.569, −0.168)	0.0006
**Smoking**	−0.115 (−0.339, 0.122)	0.3271	−0.077 (−0.306, 0.159)	0.5073
**Alcohol consumption**	−0.0134 (−0.246, 0.221)	0.9093	0.027 (−0.208, 0.259)	0.8197
**Physical activity**	−0.210 (−0.423, 0.025)	0.0704	−0.313 (−0.509, −0.086)	0.0062
**Fast food consumption**	0.254 (0.022, 0.460)	0.0281	0.240 (0.007, 0.449)	0.0377
**Meat**	0.263 (0.031, 0.467)	0.0228	0.262 (0.030, 0.467)	0.0233
**Sweet drinks**	0.061 (−0.175, 0.290)	0.6052	0.027 (−0.208, 0.259)	0.8186
**Fruits and vegetables**	0.257 (0.025, 0.463)	0.0259	0.339 (0.115, 0.531)	0.0029

A multiple logistic regression analysis was performed to investigate the influence of various factors on CRC diagnosis using age, BMI, history of malignancy, current smoking status, consumption of sweet drinks, expression of miR-29a, and expression of miR-92a (Table 4). The AUC of the ROC curve was 0.991, and sensitivity and specificity were 95.35 % and 96.88 %, respectively (Fig. 4B).

**Table 4:** Multiple logistic regression analysis of the characteristics and CRC.

**Characteristics**	**OR**	***p*-value**
Age	1.171	0.1393
BMI	1.404	0.4004
History of malignancy	57,762	0.1036
Current smoking status	0.015	0.0994
Sweet drinks	0.056	0.0971
Expression of miR-29a	0.063	0.0374
Expression of miR-92a	1.171	0.1393

### Machine learning results

To assess the effectiveness of miR-29a and miR-92a, besides other extracted features, in predicting CRC, we implemented multiple logistic regression and multilayer feedforward neural network methods. In the multiple logistic regression method, we used BMI, history of malignancy, current smoking status, sweet drink consumption, expression of miR-29a, and expression of miR-92a (mentioned in Table 4) as input features that lead to the best result, whereas in the neural network method, we utilized all extracted features including miR-29a and miR-92a, as well as demographic, clinical, and food intake data. The ROC curve analysis of the two models is depicted in Fig. 4B with an AUC of 0.980 and sensitivity and specificity of 88.89 % and 86.67 %, respectively.

## Discussion

CRC is one of the leading causes of cancer death globally, and diagnosis of CRC in its early stages plays an important role in reducing mortality. In recent years, the assessment of circulating biomarkers in CRC patients has been introduced as a diagnostic aid [29,30]. Exosomes isolated from circulating body fluids contain multiple nucleic acids, proteins, and miRNAs [31]. They are secreted by metastatic tumors and can communicate between the primary and the metastatic tumor. Several studies showed that exosomes can activate target cells and transmit surface receptors [32]. miRNAs are used in biomarker research and have been introduced for therapeutic aims. One of the advantages of miRNAs is their stability; thus, they can be detected in saliva. The present study evaluates salivary exosomal miRNAs in CRC patients. mRNA and miRNA are more stable molecules than proteins, and they are more likely to be found in saliva [33]. However, these molecules are carried by exosomes and thus are protected against degradation. Salivary gland cells secrete exosome-like microvesicles that contain proteins and mRNAs [34]. Exosome-like microvesicles derived from cancers activate transcription and translation in salivary gland cells, altering the composition of salivary gland exosome-like microvesicles as well as mRNa and protein levels [35,36].

The epithelial-to-mesenchymal transition (EMT) is a critical factor that involves multiple metabolic alterations [37,38]. miRNAs are expected to play an important role in colorectal carcinogenesis because they control EMT by targeting E-cadherin and other molecules [39,40]. Overexpression of miR-29a increases cell invasion by suppressing E-cadherin expression [41]. In addition, miR-92a plays a significant role in the development and metastasis of CRC. Shi et al. revealed the high diagnostic accuracy of plasma miR-9a in CRC. Based on their study, miR-92a is up-regulated in CRC and correlates with malignant tumor development and progression. Serum miR-92a-1 is a novel diagnostic biomarker for CRC [42]. Hassan et al. revealed up-regulation of plasma miR-21 and miR-92a in CRC compared with irritable bowel syndrome, ulcerative colitis, and healthy subjects. Their study showed that plasma miR-92a can be introduced as a potential noninvasive biomarker for CRC and differentiates between colorectal disorders. Previous studies has investigated the potential role of miR-21 and miR-92a in plasma for the diagnosis of irritable bowel syndrome, ulcerative colitis, and CRC [43]. Up-regulation of miR-92a has been reported in other studies. In addition, miR-92a is a valuable biomarker for assessing disease prognosis [44]. miR-92a promotes the invasion and migration of CRC through the RECK-MMP signaling pathway, and the up-regulation of miR-92a is associated with poor long-term prognosis in CRC [45]. Previous studies showed that miR-29a is up-regulated in CRC tissues compared with normal tissues. Mo et al. showed that serum miR-29a-3p levels were inversely related to the size of colorectal lesions [46]. Huang et al. showed that miR-92a was overexpressed in plasma CRC patients [23]. The sensitivity and specificity of miR-92a in CRC diagnosis were 85.3 % and 93 %, respectively, and the sensitivity and specificity of miR-29a in CRC diagnosis were 88 % and 93 %, respectively. These values indicate that these biomarkers may represent a novel screening biomarker for the early diagnosis of CRC. The role of miR-29a and miR-92a in CRC early diagnosis has been stated in several studies. Fukada et al. stated that miR-21 and miR-29a decreased significantly in plasma of CRC patients versus controls, and miR-29a and miR-92a could predict prognosis [47]. According to Liu et al., the mixture of miR-29a and miR-92a exhibited a high diagnostic value and an acceptable AUC [48]. Lv et al. suggested that miR-92a plays a key function in colorectal carcinogenesis by boosting CRC cell proliferation and migration [49].

Several studies on expression of miR-92a in biofluid samples of CRC patients were assessed [50,51]. Some studies investigated the role of miR-29a in patients with colorectal neoplasia [52,53]. Their findings indicate that miR-29a and miR-92a of plasma have great promise for noninvasive CRC detection [23,54]. Rapado-Gonzalez et al. confirmed five up-regulated miRNAs to be significantly higher in the CRC group than in the healthy group [55]. Sazanov et al. suggested that miR-21 in plasma and saliva could be a valuable biomarker for screening of CRC. The sensitivity and specificity of miR-21 expression were respectively estimated to be 65 % and 85 % in plasma, and 97 % and 91 % in saliva [56]. Our results showed that the sensitivity and specificity of miR-29a expression in saliva were 88 % and 93 % (*p* < 0.001), and they were 85.3 % and 93 % for miR-92a (*p* < 0.001). Both miRNAs were shown to be able to differentiate CRC patients from controls based on ROC curve analyses, with an AUC of 0.956 for miR-29a (95 % CI: 0.905–1.000) and 0.926 for miR-92a (95 % CI: 0.862–0.990). The positive predictive value of miR-29a in CRC diagnosis was 94 %. However, miR-29a was down-regulated in the other studies. So taken together, the expression pattern of miR-29a in CRC is still debatable, although it may be a marker of malignancy in CRC with some clinical relevance.

In this study, besides the statistical analysis of salivary miR-92a and miR-29a levels, machine learning techniques revealed that salivary miR-92a and miR-29a levels could be used as a noninvasive method for the early detection of CRC. In addition, it was demonstrated that diagnostic accuracy could be enhanced by simultaneously including demographic and clinical characteristics.

## Conclusion

Due to the increased expression of miR-29a and miR-92a biomarkers in salivary samples of CRC patients compared with healthy controls and the high values of accuracy for these two biomarkers, salivary levels of miR-92a and miR-29a may be accurate in CRC diagnosis. Statistical analysis and machine learning might develop cost-effective, noninvasive methods with acceptable accuracy for CRC diagnosis.

## Ethics approval and consent to participate

This study was approved by the Tehran University of Medical Sciences Ethical Committee (ethical code: IR.TUMS.DENTISTRY.REC.1399.084): https://ethics.research.ac.ir/EthicsProposalView.php?&code=IR.TUMS.DENTISTRY.REC.1399.084 https://ethics.research.ac.ir/ProposalCertificateEn.php?id=149230&Print=true&NoPrintHeader=true&NoPrintFooter=true&NoPrintPageBorder=true&LetterPrint=true

## Statement of informed consent/statement of human rights

After describing the study objectives, all participants signed the informed consent before participating in this study.

## Consent for publication

Not applicable.

## Availability of data and materials

The datasets used and/or analyzed during the current study are available from the corresponding author on reasonable request.

## CRediT authorship contribution statement

**Maryam Koopaie:** Conceptualization, Methodology, Project administration, Supervision, Visualization, Writing – original draft, Writing – review & editing. **Soheila Manifar:** Data curation, Funding acquisition, Investigation, Supervision, Visualization. **Mona Mohammad Talebi:** Conceptualization, Data curation, Funding acquisition, Investigation, Methodology, Resources, Writing – original draft. **Sajad Kolahdooz:** Formal analysis, Software, Validation, Visualization, Writing – original draft, Writing – review & editing. **Amirnader Emami Razavi:** Data curation, Methodology, Supervision, Validation, Visualization. **Mansour Davoudi:** Investigation, Software, Supervision, Validation. **Sara Pourshahidi:** Investigation, Validation, Visualization.

## Declaration of competing interest

The authors declare that they have no competing interests.
